# Parallel selection on gene copy number variations through evolution of three-spined stickleback genomes

**DOI:** 10.1186/1471-2164-15-735

**Published:** 2014-08-29

**Authors:** Shotaro Hirase, Haruka Ozaki, Wataru Iwasaki

**Affiliations:** Center for Earth Surface System Dynamics, Atmosphere and Ocean Research Institute, the University of Tokyo, Kashiwa, Chiba, 277-8564 Japan; Department of Computational Biology, Graduate School of Frontier Sciences, the University of Tokyo, Kashiwa, Chiba, 277-8568 Japan; Department of Biological Sciences, Graduate School of Science, the University of Tokyo, Bunkyo-ku, Tokyo, 113-0032 Japan

**Keywords:** Gene copy number variations, Parallel evolution, Positive selection, Three-spined stickleback

## Abstract

**Background:**

Understanding the genetic basis of adaptive evolution is one of the major goals in evolutionary biology. Recently, it has been revealed that gene copy number variations (GCNVs) constitute significant proportions of genomic diversities within natural populations. However, it has been unclear whether GCNVs are under positive selection and contribute to adaptive evolution. Parallel evolution refers to adaptive evolution of the same trait in related but independent lineages, and three-spined stickleback (*Gasterosteus aculeatus*) is a well-known model organism. Through identification of genetic variations under parallel selection, i.e., variations shared among related but independent lineages, evidence of positive selection is obtained. In this study, we investigated whole-genome resequencing data from the marine and freshwater groups of three-spined sticklebacks from diverse areas along the Pacific and Atlantic Ocean coastlines, and searched for GCNVs under parallel selection.

**Results:**

We identified 24 GCNVs that showed significant differences in the numbers of mapped reads between the two groups, and this number was significantly larger than that expected by chance. The derived group, i.e., freshwater group, was typically characterized by larger gene-copy numbers, which implied that gene duplications or multiplications helped with adaptation to the freshwater environment. Some of the identified GCNVs were those of multigenic family genes, which is consistent with the theory that fatal effects due to copy-number changes of multigenic family genes tend to be less than those of single-copy genes.

**Conclusion:**

The identification of GCNVs that were likely under parallel selection suggests that contribution of GCNVs should be considered in studies on adaptive evolution.

**Electronic supplementary material:**

The online version of this article (doi:10.1186/1471-2164-15-735) contains supplementary material, which is available to authorized users.

## Background

Understanding the genetic basis of adaptive evolution is one of the major goals in evolutionary biology [1–5]. When populations adapt to new environments, positive selection can increase frequencies of specific genetic variations that have greater fitness than others, sometimes resulting in the fixation of those variations [1–3]. To detect positive selection, two major approaches have achieved significant success. One approach is molecular evolutionary analysis of protein-coding gene sequences. Comparison of the synonymous and nonsynonymous nucleotide substitution rates has been adopted by many studies to identify positive selection [1, 6]. While this approach is applicable to only protein-coding genes that have accumulated sufficient numbers of nucleotide substitutions, the other approach targets shorter time-scale events by detecting the fixation of single nucleotide variations (SNVs) within populations [1]. Many SNVs were found to be associated with phenotypic variations, including *cis*-elemental SNVs that affect gene expression levels (e.g., [7]). Analyses of polymorphism distributions have revealed positive selection of a number of SNVs (e.g., [8, 9]).

These approaches focused on positive selection on variations due to nucleotide substitutions. However, it has recently been revealed that copy number variations (CNVs), or gains or losses of DNA segments, constitute a significant proportion of genomic diversity [10–15]. Because CNVs are known to result in significant phenotypic effects that include human diseases [16], they are also expected to be under positive selection. In particular, gene copy number variations (GCNVs), which change the numbers of gene loci in genomes, can significantly alter gene functions and dosages [17, 18]. As expected, the possibility of fixation of CNVs by positive selection has been reported in several phylogenetic groups [19, 20].

Parallel evolution, which is the adaptive evolution of the same trait in related but independent lineages, can provide evidence of positive selection, because genetic drift is unlikely to produce concerted changes in independent lineages [21]. The marine and freshwater phenotypes of three-spined sticklebacks (*Gasterosteus aculeatus*) are an excellent system to investigate parallel evolution [21]. This species inhabits a large number of marine, estuarine, and freshwater environments in Asia, Europe, and North America. After the retreat of Pleistocene glaciers, the marine ancestors have colonized and adapted to newly created freshwater habitats over the world, showing repeated changes in the body shape, skeletal armor, trophic specialization, pigmentation, salt handling, life history, and mating preference [22, 23]. Previous studies revealed that this independent evolution of similar phenotypes in the freshwater groups occurred due to *parallel selection* on the globally shared, standing SNVs in the same genes in different freshwater populations, providing strong evidence that positive selection on these SNVs contributed to the adaptive evolution toward the freshwater environments [24–26]. Recently, Feulner et al. [27] reported a significant number of CNVs in a marine population of the sticklebacks. Therefore, as with SNVs, GCNVs can also be under parallel selection through the evolution of sticklebacks. To investigate this possibility, we analyzed whole-genome resequencing data from marine and freshwater groups of three-spined sticklebacks and searched for GCNVs that contributed to the parallel evolution of the three-spined sticklebacks.

## Results and discussion

### GCNVs that likely contributed to the parallel evolution of three-spined sticklebacks

**Figure 1:**
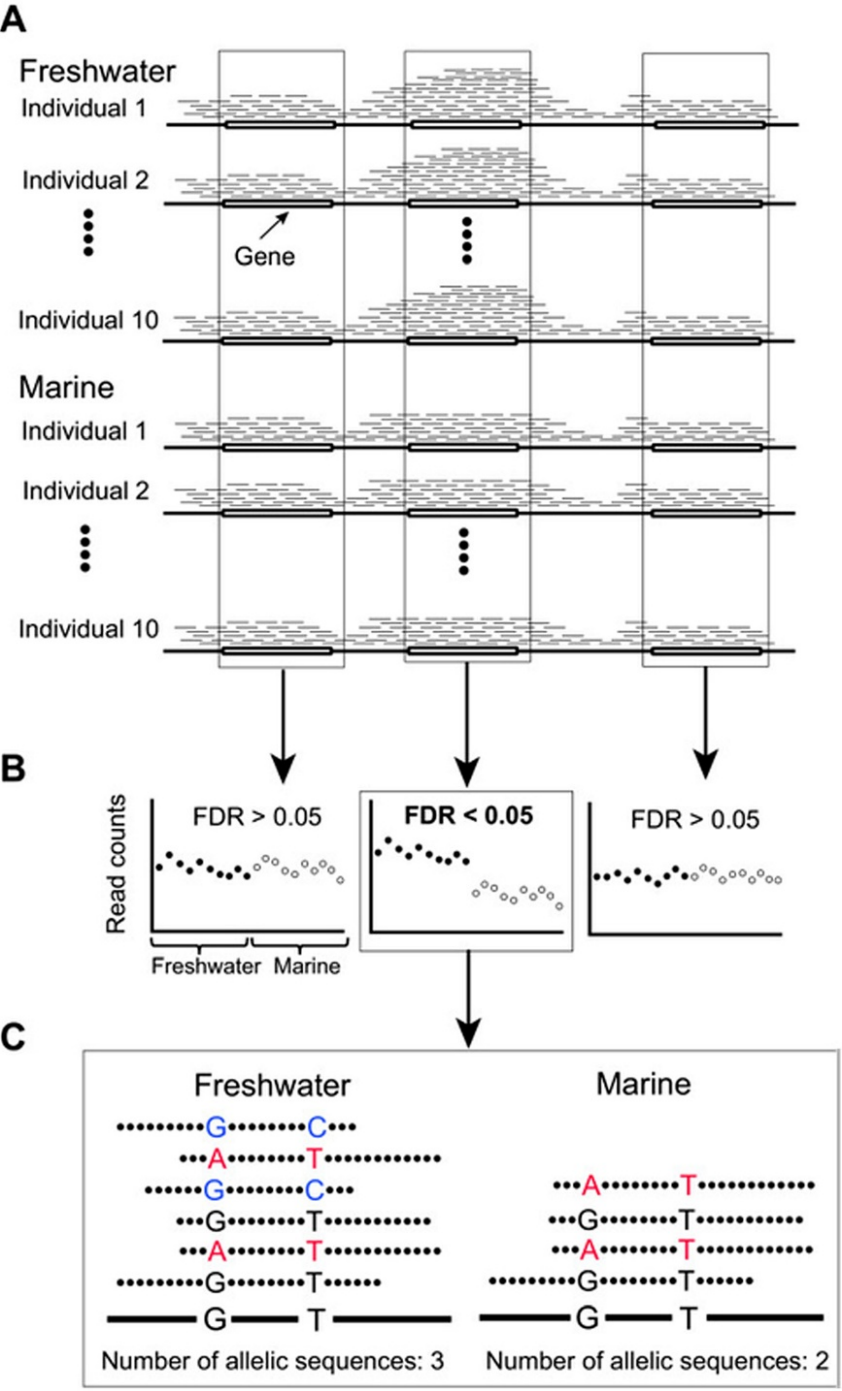
**Schematic diagram of the method for identifying GCNVs likely under parallel selection. (A)** Re-sequenced reads (thin lines) from each individual were mapped to the stickleback reference genome (thick lines). **(B)** The numbers of mapped reads that overlapped with genes were counted, and we searched for genes that showed significant differences in the normalized read numbers between the freshwater (closed circles) and marine groups (open circles) with a false discovery rate (FDR) < 0.05. Genes that showed significant differences under the three mapping options were regarded as GCNVs likely under parallel selection. **(C)** The number of different allelic sequences was counted for each of the identified GCNVs by enumerating every pair of SNV positions that was located within the read length. If three or more allelic sequences were observed for a gene, the GCNV involved duplications or multiplications.

We downloaded whole-genome resequencing data of 10 marine and 10 freshwater individuals of three-spined sticklebacks (Jones et al. [26]) from NCBI Sequence Read Archive (SRA, [28]). Both groups consisted of individuals that were derived from diverse areas along the Pacific and Atlantic Ocean coastlines (Additional file 1: Table S1). Thus, genetic variations that were specifically shared among individuals in the freshwater (and marine) group were likely due to parallel selection. To increase the sensitivity of detecting GCNVs under parallel selection, we devised a novel approach that was based on a statistical method (Figures 1A and 1B). The sequenced reads from each of the 20 individuals were mapped to the reference stickleback genome, and the numbers of the mapped reads were counted for each gene to estimate changes in their copy numbers. Genes that showed significant differences in the numbers of mapped reads between both groups were identified as GCNVs likely under parallel selection (Figures 1A and 1B; See Methods).

**Figure 2:**
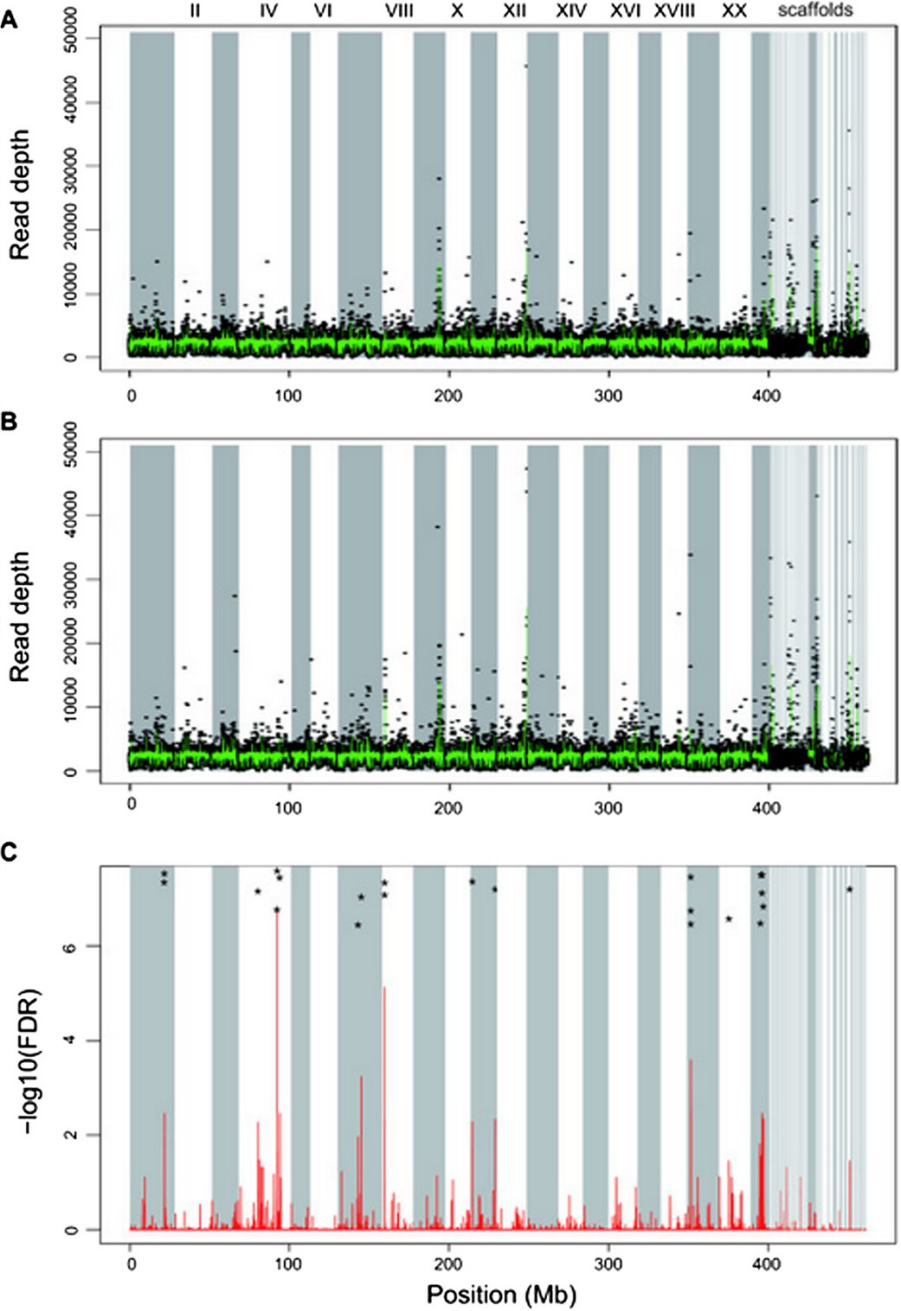
**GCNVs likely under parallel selection.** The normalized numbers of mapped reads per 1-Mb gene length for each gene across the genomes of the **(A)** freshwater and **(B)** marine groups. Each black point represents the number for each gene in each individual, and the green lines represent the mean values for each gene across individuals. **(C)** The false discovery rate of the EdgeR analysis on the differences in the numbers of mapped reads between the freshwater and marine groups for each gene. Asterisks indicate the positions of the GCNVs under parallel selection (FDR < 0.05).

Twenty-four genes showed significant differences in the numbers of mapped reads between both groups (Figure 2 and Table 1). Among these genes, five showed more copies in the individuals of the marine group (freshwater-decreased GCNVs) and 19 showed more copies in those of the freshwater group (freshwater-increased GCNVs). We confirmed that the number of the identified GCNVs was significantly larger than that expected by chance based on a permutation test (*p* < 0.05) for each mapping option. Collectively, these results suggested that the 24 GCNVs were likely due to parallel selection. Note that the 2.3× coverage of the resequencing data [26] would have led to underestimation of the numbers of GCNVs between the marine and freshwater groups. A higher sequencing coverage may result in detection of more GCNVs.

**Table 1:** **Gene copy number variations likely under parallel selection**

Ensembl gene ID	Genomic location			Group having more copies	In divergent regions [ [26]]	Gene annotation
	Linkage group	Start	End			
ENSGACG00000014268	groupI	21,543,442	21,565,537	Freshwater	Yes	Tensin 1 (TNS1)
ENSGACG00000014289	groupI	21,600,545	21,614,802	Freshwater	Yes	Serine/threonine kinase 11 interacting protein (STK11IP)
ENSGACG00000018214	groupIV	11,925,723	11,934,224	Freshwater	No	Kinesin family member 3A (KIF3A)
ENSGACG00000019313	groupIV	23,928,955	23,953,125	Freshwater	No	Tubulin tyrosine ligase-like family member 12 (TTLL12)
ENSGACG00000019321	groupIV	23,968,608	23,982,358	Freshwater	Yes	Sulfotransferase family 4A member 1 (SULT4A1)
ENSGACG00000020171	groupVII	12,721,951	12,727,083	Freshwater	No	Protein phosphatase 1 regulatory (inhibitor) subunit 14A (PPP1R14A)
ENSGACG00000014553	groupXI	15,607,308	15,613,431	Freshwater	No	Apolipoprotein L 2 (APOL2)
ENSGACG00000002886	groupXIX	2,446,925	2,473,806	Freshwater	Yes	NLR family CARD domain containing 5 (NLRC5)
ENSGACG00000002902	groupXIX	2,484,537	2,497,605	Freshwater	Yes	*Myosin heavy chain (MyHC)
ENSGACG00000002933	groupXIX	2,501,529	2,511,962	Freshwater	Yes	*Myosin heavy chain (MyHC)
ENSGACG00000006397	groupXX	6,176,973	6,190,798	Freshwater	No	Dopa decarboxylase (aromatic L-amino acid decarboxylase)(DDC)
ENSGACG00000002551	groupXXI	5,808,646	5,870,440	Freshwater	No	*Rab effector MyRIP-like (MYRIP)
ENSGACG00000002682	groupXXI	6,189,464	6,240,135	Freshwarer	No	Neuropilin (NRP) and tolloid (TLL)-like 1 (NETO1)
ENSGACG00000002744	groupXXI	6,534,938	6,558,550	Freshwater	No	Junctophilin 1 (JPH1)
ENSGACG00000002857	groupXXI	7,179,938	7,191,684	Freshwater	No	Carboxypeptidase A6 (CPA6)
ENSGACG00000002913	groupXXI	7,252,896	7,262,425	Freshwater	No	Minichromosome maintenance domain containing 2 (MCMDC2)
ENSGACG00000002918	groupXXI	7,255,256	7,257,350	Freshwater	No	*Unknown
ENSGACG00000003408	groupXXI	7,994,019	7,996,973	Freshwater	No	*Neoverrucotoxin
ENSGACG00000015099	scaffold_68	405,524	407,382	Freshwater	No	LSM14B SCD6 homolog B (S. cerevisiae) (LSM14B)
ENSGACG00000019508	groupIV	25,553,051	25,563,391	Marine	No	Neurexophilin and PC-esterase domain family member 3 (NXPE3)
ENSGACG00000020238	groupVII	14,778,775	14,788,878	Marine	No	*Gap-Pol polyprotein-like
ENSGACG00000003374	groupVIII	1,526,335	1,528,158	Marine	No	*Unknown
ENSGACG00000003379	groupVIII	1,528,722	1,530,746	Marine	No	*Unknown
ENSGACG00000005313	groupXI	1,204,843	1,206,464	Marine	No	*Heat shock protein (HSP)

*Gene annotations were based on BlastX search if Ensembl annotations were unavailable.

Among the identified GCNVs, *neurexophilin and PC-esterase domain family member 3* (*NXPE3*) overlapped with a region that was reported as a CNV in a marine group of three-spined sticklebacks [27]. In addition, the identified GCNVs included well-known multigenic families such as *sulfotransferase* (*SULT*), *NOD-like receptor* (*NLR*), *apolipoprotein L* (*APOL*), *kinesin family* (*KIF*), and *myosin heavy chain* (*MyHC*). The finding that the identified GCNVs included genes in multigenic families was consistent with the idea that GCNVs of multigenic family genes are more likely to occur than those of single-copy genes. This is because, fatal effects due to copy-number changes of multigenic family genes tend to be less than those of single-copy genes [29]. It would be notable that GCNVs were previously observed for *APOL* [30], *KIF* [31] and *SULT* [32] in primates and for *MyHC* in fish [33].

### Segmental duplications/multiplications or deletions behind the identified GCNVs

**Figure 3:**
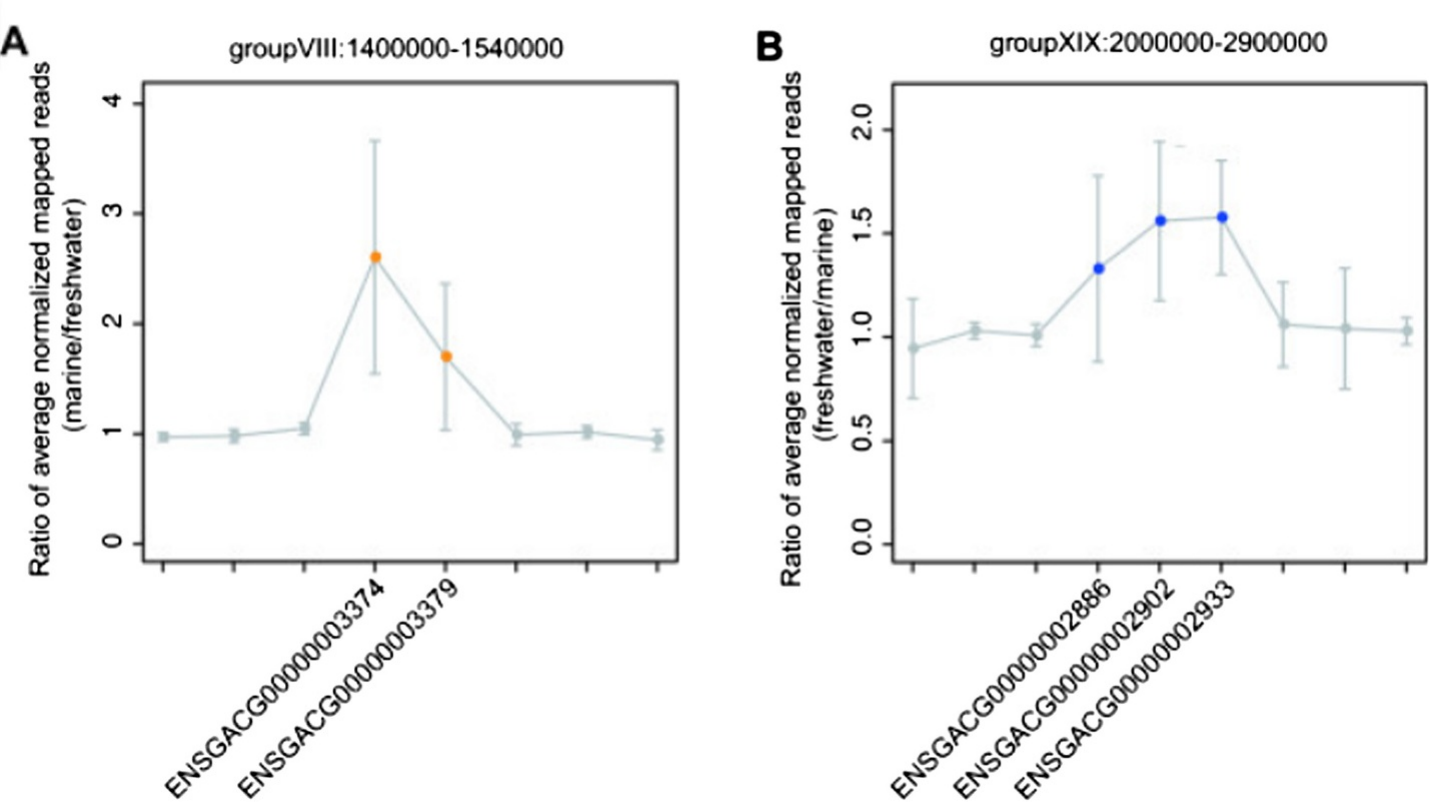
**Segmental duplications/multiplications or deletions underlying the clusters of GCNVs likely under parallel selection.** Gene clusters that included GCNVs likely under parallel selection located in the linkage groups **(A)** VIII and **(B)** XIX are shown with three genes upstream or downstream. Each point represents the ratio of the average of the normalized numbers of the mapped reads between the two groups. The identified GCNVs with more copies in the marine and freshwater groups are colored by orange and blue, respectively. Genes were excluded from visualization if the median of the numbers of mapped reads per 100 bp of the gene length was less than one or if no reads were mapped in at least one individual. The error bars indicate standard deviations of the ratios that were calculated for pairs of freshwater and marine groups derived from the same geographic regions. (If multiple samples were derived from the same geographic region for either group, the average of the normalized number of reads was used for the calculation).

An important characteristic of the 24 GCNVs likely under parallel selection was that they frequently appeared at close locations on the genomes (Figure 2). This observation implied that those GCNVs would have resulted from segmental duplications/multiplications or deletions of genomic regions that contained multiple genes (i.e., gene clusters). Figure 3 represents the ratios of the numbers of reads that were mapped to genes in and around the gene clusters in the linkage groups VIII and XIX, which were suspected to have experienced segmental duplications or deletions. This observation was consistent with a previous study that reported that CNVs sometimes involve segmental duplications [20].

Next, we compared the locations of the 24 GCNVs with *divergent regions* that were designated by Jones et al. [26], because a previous study reported that many CNVs in primates overlapped with genes under positive selection [34]. The divergent regions were three-spined stickleback genomic regions whose sequences showed signs of parallel evolution of nucleotide variations between the marine and freshwater groups. The aforementioned gene cluster in the linkage group XIX overlapped with the divergent regions, suggesting that both nucleotide sequences and copy numbers of the genes in this region would have been under parallel selection during adaptation to the freshwater environment. However, most of the GCNVs did not overlap with the divergent regions, which suggested that their copy numbers, but not sequences, would have been under parallel selection (Table 1).

### Larger gene copy numbers in the derivative, freshwater phenotype

Among the 24 GCNVs likely under parallel selection, larger gene copy numbers were more frequently associated with the freshwater group (19 out of 24, Table 1). This was consistent with the fact that the freshwater phenotype is derivative, because increase, rather than decrease, in gene copy numbers is expected to facilitate adaptation to new environments by introducing new physiology and morphology to the organism [35]. For example, Chen *et al*. suggested that duplications of protein coding genes contributed to the physiological fitness of Antarctic notothenioids in freezing polar conditions [18]. In particular, the freshwater-increased GCNVs included two genes involved in the inflammatory response (*APOL2*, *NLRC5*) and two genes that were homologous to *MyHC* (ENSGACG00000002902, ENSGACG00000002933). A previous study showed parallel divergences between littoral and pelagic phenotype pairs of three-spined stickleback *MHC* genes, which are key genes in the immune system and would be associated with parasite communities in each habitat [36]. Various types of myosin genes were reported to have appeared during the evolution of teleost fish, and those variations were supposed to have contributed to the adaptation to variable aquatic conditions [33]. Thus, we expect that those GCNVs would have played important roles in adaptation to the freshwater environment.

The larger gene copy numbers in the freshwater group could be due to the choice of the reference genome sequence. We used the reference genome that was generated from a freshwater lineage, thus the mapping efficiency of the sequencing data of the marine group might be lower for genes that accumulated many SNVs between the marine and freshwater groups. To examine whether the detected GCNVs were derived from the mapping efficiency bias toward the freshwater group, we investigated the frequencies of SNVs of the 19 freshwater-increased GCNVs using reads that were mapped with the ‘-e 100’ option. The most divergent gene was ENSGACG00000015099, which contained an average of 1.02 SNVs per 1 kb along the gene body in the marine group. This frequency was insufficient to produce the observed differences in the numbers of mapped reads. Therefore, the mapping efficiency bias was unlikely to explain the large number of the freshwater-increased GCNVs.

### GCNVs likely due to duplications or multiplications

**Figure 4:**
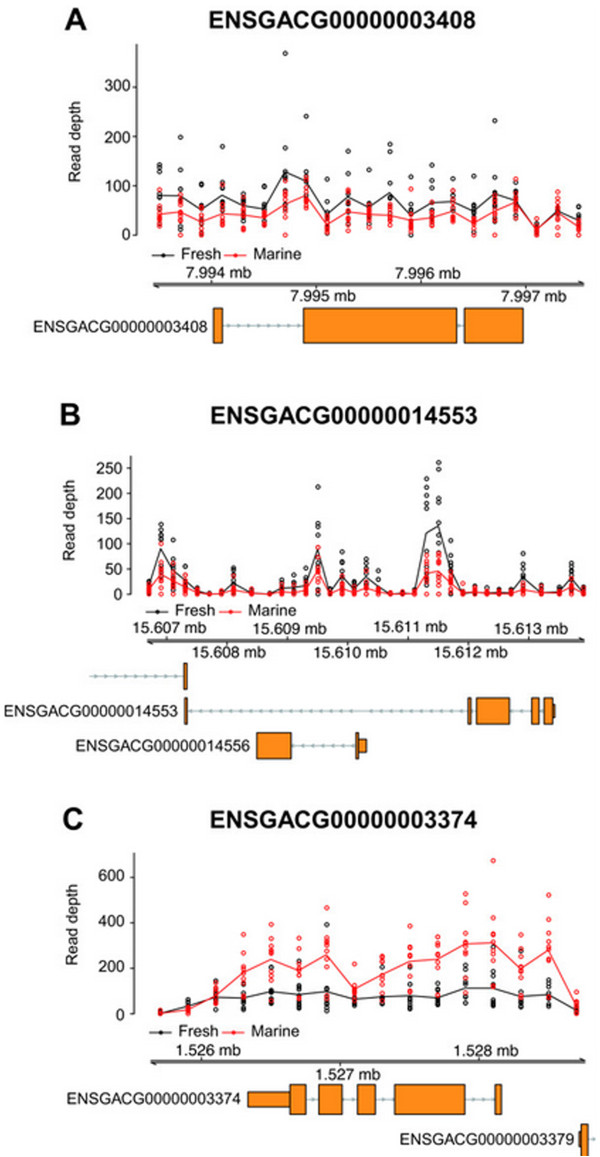
**Numbers of mapped reads in two freshwater-increased and one freshwater-decreased GCNVs.** Each point and line represent the normalized numbers and average normalized numbers, respectively, of the mapped reads per 200-bp non-overlapping window for 10 freshwater (black) and 10 marine (red) individuals. **(A and B)** Two freshwater-increased and **(C)** one freshwater-decreased GCNVs that were confirmed by three or more different allelic sequences, are shown. Gene models are shown at the bottom of each panel.

To confirm whether the detected GCNVs under parallel selection were due to duplications or multiplications in the freshwater group, we counted the numbers of different allelic sequences within the regions of the GCNVs (Figure 1C). Two freshwater-increased GCNVs (ENSGACG00000003408 and *APOL2*) (Figures 4A and B) were strongly predicted to be such GCNVs, because they were supported by at least two within-read-length SNV position pairs in three individuals of the freshwater group (Table 1 and Additional file 2: Table S2). Read depths along the genomic coordinates were not stable probably due to sequencing biases, thus their differences were clearly observed in the regions with large read depths. It was notable that the read depths in the intronic regions of *APOL2* of the freshwater group were higher than those of the marine group (Figure 4B), suggesting that this gene was recently duplicated with their intronic sequences. In addition, multiple copies of one freshwater-decreased GCNV (ENSGACG00000003374) (Figure 4C) were predicted to exist on the genomes of the marine group by the same analysis on the marine group. Another freshwater-decreased GCNV (*NXPE3*) was also supported by at least one within-read-length SNV position pair in three individuals of the marine group (Table 1 and Additional file 2: Table S2). The copy numbers of these two genes would have decreased during the adaptation to the freshwater environment.

The *APOL2* gene is a member of the *apolipoprotein L* gene family. This gene family is involved in pathogen immunity and was previously reported to have been under positive selection in primates [37]. Another previous study found copy number differences in the *APOL1* gene between human and chimpanzee and suggested that these differences were involved in the adaptive phenotype differentiation of the inflammatory response [30]. The duplications or multiplications of *APOL2* might have contributed to adaption of the immune system to the freshwater environment. For ENSGACG00000003408, we conducted BLASTX searches against NCBI nr database because no functional descriptions were available in the Ensembl database. The best hit for this gene was a neoverrucotoxin subunit alpha-like gene of *Oreochromis niloticus* with E-value = 0.0 (Accession numbers of the hits were XP_003449498, XP_003449506, and XP_003449483). This gene was reported to be overexpressed in the brooding tissue of pregnant specimens of a species in genus *Syngnathus* [38], which belongs to the same order as the three-spined stickleback does. The duplications or multiplications of ENSGACG00000003408 might have had roles in pregnancy functions in the freshwater environment. We could not obtain any hit for ENSGACG00000003374. A previous study reported GCNVs of *NXPE3* within marine populations [27]. *NXPH3* is a neuropeptide-like molecule that functions in brain [39], and neuropeptides were suggested to control migratory behaviors [40]. The decrease of the *NXPE3* copy numbers in the freshwater group might have been associated with their anadromous behavior [22].

### Differential expressions of genes between the two environments

If the two strongly supported freshwater-increased GCNVs actually contributed to the parallel evolution of the three-spined sticklebacks, the amount of transcription products of these genes should be important for the adaptation. Thus, we analyzed microarray data of gills of three-spined sticklebacks in marine and freshwater groups under the short and long photoperiod conditions [41], and evaluated whether these two genes showed significant differential expressions between the two groups. As expected, the gene expression values of *APOL2* and ENSGACG00000003408 were higher in the freshwater group than those in the marine group highly significantly (*p* < 0.005 after Bonferroni correction) under the short photoperiod condition. The short photoperiod condition resembled winter, thus these genes might have contributed to the fitness though the overwinter survival [42].

## Conclusion

In this study, we showed the possibility that GCNVs underwent positive selection in the parallel evolution of the three-spined sticklebacks and had a role in the adaptation to the freshwater environment. It would be notable that many CNVs were found in a marine population of three-spined sticklebacks [27], which suggests the existence of globally shared, standing CNVs that can contribute to the parallel evolution within natural population. Our results suggest that the contribution of GCNVs should be considered in studies on adaptive evolution of diverse species.

## Methods

### Genome sequences

The three-spined stickleback genome sequence (BROADS1.56) and the annotated gene models were taken from the Ensembl database (release 72, [43]). The genome sequence has been generated from a line derived from a freshwater population (Bear Paw Lake, [26]).

### Resequencing data processing

A resequencing dataset of 10 marine and 10 freshwater individuals was previously generated using an Illumina Genome Analyzer II (36—51 bp, single-end), which yielded approximately sixty million reads (approximately 2.3×) per individual (Jones *et al.* [26], Additional file 1: Table S1). We downloaded the data from NCBI Sequence Read Archive (SRA, [28]). The accession numbers were SRX077979, SRX079119, SRX079120, SRX077981, SRX077982, SRX077990, SRX077978, SRX076627, SRX079121, SRX077983, SRX077984, SRX077986, SRX077980, SRX077988, SRX077989, SRX077987, SRX077991, SRX077992, SRX076626, SRX077985, SRX077993, and SRX077994.

The sequenced reads from each individual were mapped to the stickleback genome using the Bowtie 0.12.8 software [44] (Figure 1A). The Bowtie option of ‘-m 1’ was adopted to remove reads with multiple hits. In addition, to obtain reliable GCNVs that were not affected by the mapping parameter selection, we adopted three different values (70, 100, and 130) for the ‘-e’ option, which designated the maximum permitted total quality values at all mismatched positions throughout a read alignment. To avoid the effects of potential PCR duplicates, if multiple reads were aligned to the same position, all of the reads except for those with the highest mapping quality were removed using SAMtools (version 0.1.18, [45]) with the command ‘samtools rmdup -s’. The statistics for each mapping option are shown in Additional file 1: Table S1.

### Identification of GCNVs likely under parallel selection

We compared the numbers of mapped reads for each gene between the freshwater and marine groups to identify GCNVs under parallel selection (Figure 1B). If the numbers of mapped reads were significantly larger in the freshwater group, the gene would have been duplicated or multiplied specifically in the genomes of the freshwater group. If the numbers were significantly smaller, the gene would have been deleted or its copy number would have decreased.

The most 5′- and 3′- positions of each gene were retrieved from the Ensembl annotation, and the numbers of mapped reads that overlapped with the above area (i.e., any exonic or intronic region) were counted using the ‘intersectBed’ command in bedtools [46]. Because insufficient numbers of mapped reads may result in the detection of false GCNVs, we removed genes from the subsequent analysis if the median of the numbers of the mapped reads per 100 bp of the gene lengths was less than one, or if no reads were mapped in at least one individual resequencing data. For normalization, the numbers were divided by the total number of mapped reads across the genome for each individual. Then, we searched for GCNVs under parallel selection by detecting genes that showed significant differences in the normalized read numbers between the freshwater and marine groups using the edgeR package [47] with a false discovery rate (FDR) < 0.05. We regarded genes that were significant under all of the three different mapping options (“-e 70”, “-e 100”, and “-e 130”) as GCNVs likely under parallel selection.

To confirm that the number of identified GCNVs under parallel selection was significantly larger than that expected by chance (i.e., by genetic drift), we calculated an empirical *p* value based on a permutation test. We randomly reallocated the 10 freshwater and 10 marine individuals into two groups 10,000 times, performed the same analyses, and obtained the null distribution of numbers of GCNVs.

### Identification of gene duplications or multiplications

If the identified GCNVs involved gene duplications or multiplications, three or more different allelic sequences should be observed within the gene in each individual of each group, because three or more different allelic sequences cannot originate from a diploid genome. Thus, we examined whether three or more different allelic sequences were observed in the identified GCNVs (Figure 1C).

For each of the identified GCNVs, SNVs were called by applying the SAMtools/BCFtools pipeline [45] to the reads that were mapped with the ‘-e 100’ option. The SAMtools/BCFtools pipeline was used with default parameters, except for the ‘-Q 30’ option, to consider bases that were called with high quality only. We enumerated every pair of SNV positions that was located within the read length, i.e., 36 bp (*within-read-length SNV position pairs*). The numbers of different nucleotide pairs for each of the within-read-length SNV position pairs were counted, where each nucleotide pair was supported by multiple reads. Finally, we selected GCNVs that showed three or more different nucleotide pairs in at least three individuals of either group.

### Gene annotations

For each GCNV likely under parallel selection, we obtained functional annotations of the gene from the Ensembl database. If the functional annotations were unavailable, BLASTX searches [48] against the NCBI non-redundant protein database (nr) [49] were conducted with an E-value cutoff of 1e-14, and the hit with the highest bit-score and its annotated protein name was retrieved.

### Microarray data analysis

Microarray data of gills of two families of pure marine and pure freshwater crosses under short and long photoperiods [41] were downloaded from Center for Information Biology Gene Expression (http://cibex.nig.ac.jp) with the accession number CBX139. Two marine and freshwater datasets were treated as biological replicates. If multiple probes were mapped to one transcript, the median signal intensity of these probes was used. After removing intra-gene probes, genes with significant expression-value differences between the marine and freshwater groups were identified using the eBayes method in the limma package [50].

## Electronic supplementary material

Additional file 1: Table S1: Summary of the resequencing datasets of 10 marine and 10 freshwater sticklebacks. (PDF 49 KB)

Additional file 2: Table S2: Numbers of SNV pairs in which three or more allelic sequences were observed for each GCNVs. (PDF 48 KB)
