# Supplementary material for: Parallel selection on gene copy number variations through evolution of three-spined stickleback genomes
Source: BMC Genomics. 2014 Aug 29;15(1):735. doi: 10.1186/1471-2164-15-735 (PMC4159527; doi:10.1186/1471-2164-15-735)
Supplement: Supplementary file 1 — Additional file 1: Table S1: Summary of the resequencing datasets of 10 marine and 10 freshwater sticklebacks. (PDF 49 KB) [file 12864_2014_6411_MOESM1_ESM.pdf]

Table S1. Summary of resequencing dataset of 10 marine and 10 freshwater sticklebacks and mapping statistics

Population ID	Accession number	Phenotype	Basin	Gographic region	Number of reads	Number of reads aligned (Percentage)		
						-e 70	-e 100	-e 130
ABW	SRX077979	Freshwater	Atlantic	Iceland	65904900	36426325 (55.27%)	36587409 (55.52%)	36596174 (55.53%)
BIGL	SRX079119	Freshwater	Pacific	California	62982716	33091246 (52.54%)	34251500 (54.38%)	34800423 (55.25%)
FTC	SRX079120	Freshwater	Pacific	Washington	130008902	54744497 (42.11%)	54763528 (42.12%)	54762816 (42.12%)
HUTU	SRX077981, SRX077982	Freshwater	Pacific	Washington	152468755	54845476 (35.97%)	56089798 (36.79%)	56544376 (37.09%)
MATA	SRX077990	Freshwater	Pacific	California	66055414	33542818 (50.78%)	33530990 (50.76%)	33504790 (50.72%)
MUDL	SRX077978	Freshwater	Pacific	Alaska	39467325	21045530 (53.32%)	21183088 (53.67%)	21206196 (53.73%)
NOST	SRX076627	Freshwater	Atlantic	Norway	18812768	11152066 (59.28%)	11241084 (59.75%)	11285321 (59.99%)
PAXB	SRX079121	Freshwater	Pacific	British Columbia	53647262	30239994 (56.37%)	31054401 (57.89%)	31359584 (58.46%)
SCX	SRX077983	Freshwater	Atlantic	Germany	34331961	17594106 (51.25%)	17749879 (51.70%)	17779676 (51.79%)
SHEL	SRX077984	Freshwater	Atlantic	Scotland	54599491	27542876 (50.45%)	27692432 (50.72%)	27711276 (50.75%)
ANTL	SRX077986	Marine	Atlantic	Nova Scotia	40210299	14831972 (36.89%)	14997902 (37.30%)	15028743 (37.38%)
BDGB	SRX077980	Marine	Pacific	California	35332775	20949468 (59.29%)	21111998 (59.75%)	21142925 (59.84%)
BIGR	SRX077988	Marine	Pacific	California	36099888	22842437 (63.28%)	22846665 (63.29%)	22827525 (63.23%)
GJOG	SRX077989	Marine	Atlantic	Iceland	53822455	28877690 (53.65%)	29040512 (53.96%)	29077068 (54.02%)
GORT	SRX077987	Marine	Atlantic	Scotland	48046011	27148586 (56.51%)	27276293 (56.77%)	27279833 (56.78%)
JAMA	SRX077991	Marine	Pacific	Japanese	58203987	31192808 (53.59%)	32074859 (55.11%)	32443325 (55.74%)
JMRP	SRX077992	Marine	Atlantic	Scotland	56314624	20602716 (36.59%)	20608385 (36.60%)	20600818 (36.58%)
NEU	SRX076626	Marine	Atlantic	Germany	85802994	34304198 (39.98%)	34324465 (40.00%)	34318061 (40.00%)
RABS	SRX077985, SRX077993	Marine	Pacific	Alaska	65798576	32386458 (49.22%)	33019706 (50.18%)	33251296 (50.53%)
SALR	SRX077994	Marine	Pacific	British Columbia	36887238	18176089 (49.27%)	19420930 (52.65%)	20025564 (54.29%)
