# Supplementary material for: Parallel selection on gene copy number variations through evolution of three-spined stickleback genomes
Source: BMC Genomics. 2014 Aug 29;15(1):735. doi: 10.1186/1471-2164-15-735 (PMC4159527; doi:10.1186/1471-2164-15-735)
Supplement: Supplementary file 2 — Additional file 2: Table S2: Numbers of SNV pairs in which three or more allelic sequences were observed for each GCNVs. (PDF 48 KB) [file 12864_2014_6411_MOESM2_ESM.pdf]

Table S2. Numbers of SNV pairs in which three or more haplotypes were observed based on '-e 100' mapping condition

Ensembl gene ID	Group having	Freshwater										Marine									
	more copies	ABW	BIGL	FTC	HUTU	MATA	MUDL	NOST	PAXB	SCX	SHEL	ANTL	BDGB	BIGR	GJOG	GORT	JAMA	JMRP	NEU	RABS	SALR
ENSGACG00000002551	Freshwater	0	0	0	0	0	0	0	0	0	0	0	0	0	0	0	0	0	0	0	0
ENSGACG00000002682	Freshwarer	0	0	0	0	0	0	0	0	0	0	0	0	0	0	0	0	0	1	0	0
ENSGACG00000002744	Freshwater	0	0	0	3	0	0	0	0	0	0	0	0	0	0	0	0	0	2	0	0
ENSGACG00000002857	Freshwater	0	0	0	0	0	0	0	0	0	0	0	0	0	0	0	0	0	0	0	0
ENSGACG00000002886	Freshwater	0	0	0	0	0	0	0	0	0	0	0	0	0	0	0	0	0	0	0	0
ENSGACG00000002902	Freshwater	0	0	0	0	0	0	0	0	0	0	0	0	0	0	0	0	0	0	0	0
ENSGACG00000002913	Freshwater	0	0	0	0	0	0	0	0	0	0	0	0	0	0	0	0	0	0	0	0
ENSGACG00000002918	Freshwater	0	0	0	0	0	0	0	0	0	0	0	0	0	0	0	0	0	0	0	0
ENSGACG00000002933	Freshwater	0	0	0	0	0	0	0	0	0	0	0	0	0	0	0	0	0	0	0	0
ENSGACG00000003408	Freshwater	0	0	1	6	1	0	0	3	0	0	0	0	2	0	0	0	0	0	0	0
ENSGACG00000006397	Freshwater	0	0	0	0	0	0	0	0	0	0	0	0	0	0	0	0	0	0	0	0
ENSGACG00000014268	Freshwater	0	0	0	0	0	0	0	0	0	0	0	0	0	0	0	0	0	0	0	0
ENSGACG00000014289	Freshwater	0	0	0	7	0	0	0	0	0	0	0	0	0	0	0	0	0	0	0	0
ENSGACG00000014553	Freshwater	0	4	3	1	5	0	0	3	0	0	0	0	0	0	0	1	0	0	0	0
ENSGACG00000015099	Freshwater	0	0	0	0	0	0	0	0	0	0	0	0	0	0	0	0	0	0	0	0
ENSGACG00000018214	Freshwater	0	0	0	0	0	0	0	0	0	0	0	0	0	0	0	0	0	0	0	0
ENSGACG00000019313	Freshwater	0	0	0	0	0	0	0	1	0	0	0	0	0	0	0	0	0	0	0	0
ENSGACG00000019321	Freshwater	0	0	0	0	0	0	0	0	0	0	0	0	0	0	0	0	0	0	0	0
ENSGACG00000020171	Freshwater	0	0	0	0	0	0	0	0	0	0	0	0	0	0	0	0	0	0	0	0
ENSGACG00000003374	Marine	1	0	0	0	0	0	1	3	0	1	0	0	0	10	4	6	1	3	9	1
ENSGACG00000003379	Marine	0	0	0	0	0	0	0	0	0	0	0	0	0	1	0	0	0	0	0	0
ENSGACG00000005313	Marine	0	0	1	0	0	0	0	0	0	0	0	0	0	0	0	0	0	0	0	0
ENSGACG00000019508	Marine	0	0	0	1	0	0	0	0	0	0	0	0	1	0	0	1	0	0	4	0
ENSGACG00000020238	Marine	0	0	0	3	0	0	0	0	0	0	0	0	1	0	0	0	0	0	3	0
